# The threshold of alpha-fetoprotein (AFP) for the diagnosis of hepatocellular carcinoma: A systematic review and meta-analysis

**DOI:** 10.1371/journal.pone.0228857

**Published:** 2020-02-13

**Authors:** Jiaxin Zhang, Guang Chen, Peng Zhang, Jiaying Zhang, Xiaoke Li, Da’nan Gan, Xu Cao, Mei Han, Hongbo Du, Yong’an Ye

**Affiliations:** 1 Dongzhimen Hospital, Beijing University of Chinese Medicine, Beijing, China; 2 Institute of Liver Diseases, Beijing University of Chinese Medicine, Beijing, China; 3 Ministry of Education Key Laboratory of Bioinformatics, Tsinghua-Peking Center for Life Sciences, School of Life Sciences, Tsinghua University, Beijing, China; 4 Center for Evidence-based Chinese Medicine, Beijing University of Chinese Medicine, Beijing, China; Texas A&M University, UNITED STATES

## Abstract

**Objective:**

Hepatocellular carcinoma (HCC) has become a pressing health problem facing the world today due to its high morbidity, high mortality, and late discovery. As a diagnostic criteria of HCC, the exact threshold of Alpha-fetoprotein (AFP) is controversial. Therefore, this study was aimed to systematically estimate the performance of AFP in diagnosing HCC and to clarify its optimal threshold.

**Methods:**

Medline and Embase databases were searched for articles indexed up to November 2019. English language studies were included if both the sensitivity and specificity of AFP in the diagnosis of HCC were provided. The basic information and accuracy data included in the studies were extracted. Combined estimates for sensitivity and specificity were statistically analyzed by random-effects model using MetaDisc 1.4 and Stata 15.0 software at the prespecified threshold of 400 ng/mL, 200 ng/mL, and the range of 20–100 ng/mL. The optimal threshold was evaluated by the area under curve (AUC) of the summary receiver operating characteristic (SROC).

**Results:**

We retrieved 29,828 articles and included 59 studies and 1 review with a total of 11,731 HCC cases confirmed by histomorphology and 21,972 control cases without HCC. The included studies showed an overall judgment of at risk of bias. Four studies with AFP threshold of 400 ng/mL showed the summary sensitivity and specificity of 0.32 (95%CI 0.31–0.34) and 0.99 (95%CI 0.98–0.99), respectively. Four studies with AFP threshold of 200 ng/mL showed the summary sensitivity and specificity of 0.49 (95%CI 0.47–0.50) and 0.98 (95%CI 0.97–0.99), respectively. Forty-six studies with AFP threshold of 20–100 ng/mL showed the summary sensitivity and specificity of 0.61 (95%CI 0.60–0.62) and 0.86 (95%CI 0.86–0.87), respectively. The AUC of SROC and Q index of 400 ng/mL threshold were 0.9368 and 0.8734, respectively, which were significantly higher than those in 200 ng/mL threshold (0.9311 and 0.8664, respectively) and higher than those in 20–100 ng/mL threshold (0.8330 and 0.7654, respectively). Furthermore, similar result that favored 400 ng/mL were shown in the threshold in terms of AFP combined with ultrasound.

**Conclusion:**

AFP levels in serum showed good accuracy in HCC diagnosis, and the threshold of AFP with 400 ng/mL was better than that of 200 ng/mL in terms of sensitivity and specificity no matter AFP is used alone or combined with ultrasound.

## Introduction

Hepatocellular carcinoma (HCC) remains one of the most invasive cancers in humans, mostly occurring in patients with chronic liver disease, and the third leading cause of cancer-related death throughout the world [1]. Although its causes, prevention, and treatment strategies are recommended in guidelines, HCC is expected to become a pressing health problem facing the world in the coming decades [1, 2] Although researchers are making strides in HCC monitoring and treatment, there has been little improvement in survival in patients with HCC. In the United States, the 5-year survival rate of patients with HCC is still less than 12% [3]. The effective therapies are very limited for advanced HCC whose the survival rate decreased significantly [4], while there are several available treatments for the management of HCC with early stage, such as radical resection or liver transplantation, where 5-year survival rate of HCC patients who met the Milan criteria (single nodule < 5cm or three nodules diameter < 3cm) after liver transplantation was more than 70% [5, 6]. Therefore, the early discovery of HCC might be very important, and it is reported that early detection of HCC can improve the clinical outcomes [7]. Based on the evidence of benefits from early detection of HCC, the guidelines of both American Association, Asian Pacific Association, and Japan Association recommend HCC monitoring in high-risk patients for early diagnosis of HCC [8–11].

The alpha-fetoprotein (AFP) in serum is currently available diagnostic marker for HCC discovery. As for patients with chronic liver disease, a sustained increase in AFP serum level was shown to be one of the risk factors of HCC and has been used to help identify high-risk subgroup of chronic liver disease [12]. In patients with liver cirrhosis, fluctuations in AFP levels may reflect the sudden onset of viral hepatitis, the deterioration of the potential liver disease, or the development of HCC [13]. Besides, the level of AFP was reported to interact with some molecular subtypes such as EpCAM positive in invasive HCC [14–16]. It is established that multiple factors could contribute to the AFP level, which increases the difficulty of identifying the threshold. When the cutoff value of AFP was 20 ng/ml, the detection showed relatively good sensitivity with poor specificity, while when the cutoff value was 200 ng/ml, the discovery performed high specificity, but the sensitivity decreased significantly [17]. In 2001 and 2017 diagnostic staging standard of HCC in China, AFP 400 ng/mL was used as the diagnostic threshold [18]. However, a meta-analysis [19] shows that the diagnostic efficiency of AFP ≥ 200 ng/mL may be higher, partly because some of the early HCC [20] may be missed in the population with low concentration of AFP (20 to 200 ng/mL) if 400 ng/mL is still used as the criteria in HCC screening. Therefore, up to now, the optimal threshold of AFP for the diagnosis of HCC is still controversial [21–23].

In addition, it has been reported that AFP combined with ultrasound detection might improve the detection rate of HCC [24]. Both American Association for the Study of Liver Disease (AASLD) and European Association for the Study of the Liver (EASL) suggest that it is necessary to monitor HCC in high-risk patients partly by abdominal ultrasonography every six months, but there exists argument in the use of AFP as an auxiliary monitoring test and there is no identified threshold of AFP when the combination of AFP and ultrasound is used to monitor HCC [25, 26].

Therefore, it is particularly important to explore the optimal screening and diagnostic threshold of serum AFP with or without ultrasound for early diagnosis of HCC. The purpose of this study was to identify the optimal diagnostic threshold of serum AFP by systematic review and meta analysis. This article was performed based on Meta-Analysis of Observational Studies in Epidemiology (MOOSE) and reported in accordance with Preferred Reporting Items for Systematic Reviews and Meta-analysis (PRISMA) statement [27, 28], and Qualitu assessment for studies of diagnostic accuracy (QUADAS-2) was used to evaluate the quality of diagnostic test [29].

## Results

**Fig 1:**
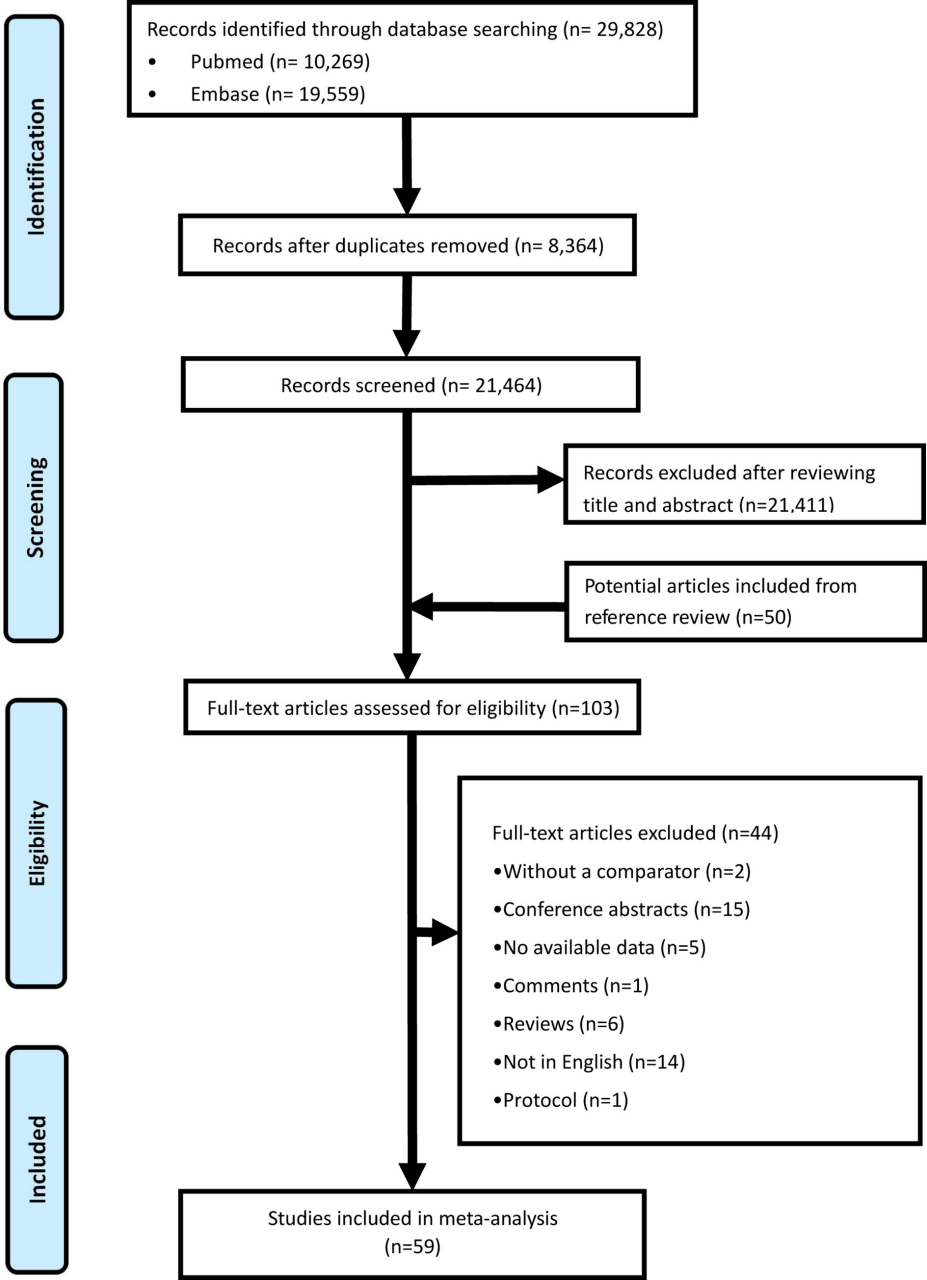
Flow diagram of study selection.

We retrieved 29,828 records from databases search, and assessed 21,464 records after deleting the duplication, and finally 59 original articles in terms of AFP alone and one systematic review in terms of AFP in combination with ultrasound [30–87] were enrolled for data synthesis, as is shown in **Fig 1**. This systematic review finally yielded information on a total of 11,731 HCC cases confirmed by histomorphology and 21,972 control cases without HCC.

### Basic information and quality assessment

The basic information of the included studies was shown in **Table 1**. In all, we summarized the results from 4 studies using a AFP threshold of 400 ng/mL, and from 4 studies using a AFP threshold of 200 ng/mL, and 46 studies using a AFP threshold of 20–100 ng/mL. As for the sample, the serum was used to detect the AFP by forty-three studies, while the remaining used plasma. The included 59 researches were conducted in diverse countries, including China (n = 15), USA (n = 11), Japan (n = 9), Korea (n = 8), Egypt (n = 5), Italy (n = 2), Thailand (n = 2), France (n = 2), South Africa (n = 1), Turkey (n = 1), India (n = 1), Germany (n = 1), Indonesia (n = 1), and Australia (n = 1). Thirty-seven studies used samples from Asian while twenty-three studies used samples from Caucasian. As for the etiology of HCC, 16 studies [49, 51, 52, 55, 57, 59, 62, 64, 66, 69, 73, 75, 79–81, 86] only covered HBV or HCV hepatitis, one study was not available, and the remaining 42 studies [30–48, 50, 53, 54, 56, 58, 60, 61, 63, 65, 68, 70–72, 74, 76–78, 82–84, 86, 87] were mix which included HBV infection, HCV infection, alcohol and others. Shown were the estimates of sensitivity, specificity, true positive, false positive, false negative, true negative in terms of AFP in HCC diagnosis in the **Table 2**. The quality assessment by QUADAS-2 tool revealed a overall judgment of at low risk of bias for the included studies, which was shown in **S3 Table**. Specifically, domain of patient selection, index test, and flow and timing showed a low risk of bias, domain of reference standard showed a conclusion of potential for bias exits, and the applicability concerns were rated as low.

**Table 1:** Characteristics of studies included in the meta-analysis.

Study	Year	Country	HCC/controls	Etiology	Assay type	Cut-off (ng/mL)	Sample type
King et al. [30]	1989	South Africa	98/120	MIX	ELISA	20	Serum
Takikawa et al. [31]	1992	Japan	116/512	MIX	ELISA	20	Plasma
Fujiyama et al. [32]	1992	Japan	200/197	MIX	ELISA	20	Plasma
Suehiro et al. [33]	1994	Japan	185/118	MIX	ELISA	20	Plasma
Grazi et al. [34]	1995	Italy	111/116	MIX	ELISA	20	Serum
Nomura et al. [35]	1999	Japan	36/49	MIX	ELISA	20	Serum
Sassa et al. [36]	1999	Japan	61/134	MIX	ELISA	20	Serum
Ishii et al. [37]	2000	Japan	29/705	MIX	ELISA	20	Serum
Cui et al. [38]	2002	China	60/30	MIX	ELISA	20	Serum
Shimizu et al. [39]	2002	Japan	56/34	MIX	ELISA	20	Serum
Cui et al. [40]	2003	China	120/90	MIX	ELISA	20	Serum
Marrero et al. [41]	2003	USA	55/104	MIX	ELISA	20	Serum
Marrero et al. [42]	2005	USA	144/108	MIX	ELISA	99	Serum
Wang et al. [43]	2005	China	61/64	MIX	ELISA	20	Serum
Kim et al. [44]	2006	Korea	62/60	MIX	CH	70.4	Plasma
Volk et al. [45]	2007	USA	84/169	MIX	ELISA	23	Serum
Durazo et al. [46]	2008	USA	144/96	MIX	ELISA	25	Serum
Beneduce et al. [47]	2008	Italy	33/31	MIX	ELISA	20	Serum
Wang et al. [48]	2009	USA	164/113	MIX	ELISA	NK	Serum
Hu et al. [49]	2009	China	31/93	HBV	ELISA	36	Serum
Marrero et al. [50]	2009	USA	419/417	MIX	ELISA	20	Serum
Yoon et al. [51]	2009	Korea	106/100	HBV	ELISA	20	Serum
Sterling et al. [52]	2009	USA	74/298	HCV	ELISA	20	Serum
Baek et al. [53]	2009	Korea	227/100	MIX	ELISA	20	Serum
Yamamoto et al. [54]	2009	Japan	190/490	MIX	ELISA	20	Serum
Mao et al. [55]	2010	China, USA	789/3428	HBV	ELISA	35	Serum
Ozkan et al. [56]	2010	Turkey	75/83	MIX	ELISA	4.36	Serum
Bessa et al. [57]	2010	Egypt	30/30	HCV	ELISA	69.5	Plasma
Sharma et al. [58]	2010	India	70/38	MIX	ELISA	13	Serum
Ishida et al. [59]	2010	Japan	141/143	HCV	ELISA	20	Serum
Tian et al. [60]	2011	China	153/219	MIX	ELISA	13.6	Serum
Shi et al. [61]	2011	China	55/107	MIX	ELISA	400	Serum
Makarem et al. [62]	2011	Egypt	113/120	HCV	CH	43	Plasma
Morota et al. [63]	2011	USA	70/34	MIX	ELISA	15	Serum
Salem et al. [64]	2012	Egypt	30/40	HCV	ELISA	10.4	Serum
Shang-1 et al. [65]	2012	Thailand	91/23	MIX	ELISA	20	Plasma
Shang-2 et al. [65]	2012	USA	40/73	MIX	ELISA	20	Plasma
Yang et al. [66]	2013	China	179/80	HBV	CH	20	Plasma
Choi et al. [67]	2013	Korea	90/78	NA	ELISA	10	Serum
Ertle et al. [68]	2013	Germany	164/422	MIX	ELISA	10	Serum
Xu-1 et al. [69]	2014	China	2472/578	HBV	ELISA	20	Serum
Xu-2 et al. [69]	2014	China	2472/578	HBV	ELISA	200	Serum
Xu-3 et al. [69]	2014	China	2472/578	HBV	ELISA	400	Serum
Chan-1 et al. [70]	2014	China	562/243	MIX	CH	10	Serum
Chan-2 et al. [70]	2014	China	562/243	MIX	CH	200	Serum
Chan-3 et al. [70]	2014	China	562/243	MIX	CH	500	Serum
Gopal-1 et al. [71]	2014	USA	452/676	MIX	ELISA	20	Serum
Gopal-2 et al. [71]	2014	USA	452/676	MIX	ELISA	200	Serum
Gopal-3 et al. [71]	2014	USA	452/676	MIX	ELISA	400	Serum
Lee et al. [72]	2014	Korea	120/40	MIX	ELISA	6	Serum
Nabih et al. [73]	2014	Egypt	35/34	HCV	CH	240	Plasma
Song et al. [74]	2014	China	550/604	MIX	ELISA	21	Serum
Costa et al. [75]	2015	France	75/75	HCV	ELISA	20	Plasma
Poté et al. [76]	2015	France	85/43	MIX	ELISA	5	Serum
Chang et al. [77]	2015	China	363/1234	MIX	ELISA	20	Serum
Gani et al. [78]	2015	Indonesia	59/47	MIX	ELISA	20.45	Serum
Chimparlee et al. [79]	2015	Thailand	157/170	HBV	ELISA	20	Serum
Fouad et al. [80]	2015	Egypt	25/25	HCV	ELISA	142	Serum
Ge et al. [81]	2015	China	89/301	HBV	ELISA	6.79	Serum
Yu et al. [82]	2015	China	134/347	MIX	CLEIA	20	Serum
Jang et al. [83]	2016	Korea	208/193	MIX	ELISA	20	Plasma
Roslyn et al. [84]	2016	Australia	86/258	MIX	CH	20	Serum
Ji et al. cohort A [85]	2016	China	236/135	HBV	ELISA	20	Serum
Ji et al. cohort B [85]	2016	China	200/97	HBV	ELISA	20	Serum
Ahn-1 et al. [86]	2016	Korea	366/366	MIX	ELISA	20	Serum
Ahn-2 et al. [86]	2016	Korea	366/366	MIX	ELISA	100	Serum
Ahn-3 et al. [86]	2016	Korea	366/366	MIX	ELISA	200	Serum
Ahn-4 et al. [86]	2016	Korea	366/366	MIX	ELISA	400	Serum
Lim et al. [87]	2016	Korea	361/276	MIX	ELISA	20	Serum

MIX: the etiology including HBV infection, HCV infection, alcohol and others; ELISA: enzyme immunometric assay; CH: chemiluminescence; CLEIA: chemiluminescence enzyme immunoassay; NK = not known; NA: not available.

**Table 2:** The indicators for HCC diagnosis were extracted from the included studies.

Study	Year	SE (%)	SP (%)	TP	FP	FN	TN
King et al. [30]	1989	74	99	73	1	25	119
Takikawa et al. [31]	1992	71	75	82	128	34	384
Fujiyama et al. [32]	1992	51	97	102	6	98	191
Suehiro et al. [33]	1994	65	72	120	33	65	85
Grazi et al. [34]	1995	55	97	61	3	50	113
Nomura et al. [35]	1999	58	76	21	12	15	37
Sassa et al. [36]	1999	8	100	5	0	56	134
Ishii et al. [37]	2000	62	78	18	155	11	550
Cui et al. [38]	2002	59	85	35	4	25	26
Shimizu et al. [39]	2002	57	63	32	13	24	21
Cui et al. [40]	2003	93	63	112	33	8	57
Marrero et al. [41]	2003	67	86	37	15	18	89
Marrero et al. [42]	2005	30	96	43	4	101	104
Wang et al. [43]	2005	59	77	36	15	25	49
Kim et al. [44]	2006	54.8	100	34	0	28	60
Volk et al. [45]	2007	62	91	52	15	32	154
Durazo et al. [46]	2008	48	87	69	12	75	84
Beneduce et al. [47]	2008	69	88	23	4	10	27
Wang et al. [48]	2009	95	21	156	89	8	24
Hu et al. [49]	2009	48	97	15	3	16	90
Marrero et al. [50]	2009	59	90	247	42	172	375
Yoon et al. [51]	2009	61	71	65	29	41	71
Sterling et al. [52]	2009	55	77	41	69	33	229
Baek et al. [53]	2009	51	91	116	9	111	91
Yamamoto et al. [54]	2009	58	88	110	59	80	431
Mao et al. [55]	2010	58	85	458	514	331	2914
Ozkan et al. [56]	2010	83	95	62	4	13	79
Bessa et al. [57]	2010	60	90	18	3	12	27
Sharma et al. [58]	2010	73	66	51	13	19	25
Ishida et al. [59]	2010	52	61	73	56	68	87
Tian et al. [60]	2011	95	47	145	116	8	103
Shi et al. [61]	2011	38	93	21	7	34	100
Makarem et al. [62]	2011	74	100	84	0	29	120
Morota et al. [63]	2011	63	91	44	3	26	31
Salem et al. [64]	2012	90	78	27	9	3	31
Shang-1 et al. [65]	2012	53	93	21	5	19	68
Shang-2 et al. [65]	2012	78	96	71	1	20	22
Yang et al. [66]	2013	37	85	66	12	113	68
Choi et al. [67]	2013	79	85	71	12	19	66
Ertle et al. [68]	2013	55	95	90	21	74	401
Xu-1 et al. [69]	2014	69.74	91.18	1724	51	748	527
Xu-2 et al. [69]	2014	51.58	97.75	1275	13	1197	565
Xu-3 et al. [69]	2014	31.47	99.13	778	5	1694	573
Chan-1 et al. [70]	2014	82.6	70.4	464	72	98	171
Chan-2 et al. [70]	2014	47.7	97.1	268	7	294	236
Chan-3 et al. [70]	2014	38.1	100	214	0	348	243
Gopal-1 et al. [71]	2014	70.1	89.8	317	69	135	607
Gopal-2 et al. [71]	2014	50	99.4	226	4	226	672
Gopal-3 et al. [71]	2014	44	99.9	199	1	253	675
Lee et al. [72]	2014	64	95	77	2	43	38
Nabih et al. [73]	2014	49	91	17	3	18	31
Song et al. [74]	2014	61	93	336	42	214	562
Costa et al. [75]	2015	49	87	37	11	38	65
Poté et al. [76]	2015	68	51	58	21	27	22
Chang et al. [77]	2015	53	93	192	83	171	1151
Gani et al. [78]	2015	73	92	43	4	16	43
Chimparlee et al. [79]	2015	67	97	105	5	52	165
Fouad et al. [80]	2015	100	100	25	0	0	25
Ge et al. [81]	2015	72	88	64	36	25	265
Yu et al. [82]	2015	77	65	103	121	31	226
Jang et al. [83]	2016	62	90	129	19	79	174
Roslyn et al. [84]	2016	43	97	37	9	49	249
Ji et al. cohort A [85]	2016	68	81	160	26	76	109
Ji et al. cohort B [85]	2016	62	69	124	30	76	67
Ahn-1 et al. [86]	2016	50.55	87.7	185	45	181	321
Ahn-2 et al. [86]	2016	37.7	95.9	138	15	228	351
Ahn-3 et al. [86]	2016	30.05	97.27	110	10	256	356
Ahn-4 et al. [86]	2016	24.04	98.36	88	6	278	360
Lim et al. [87]	2016	56.8	82.8	205	47	156	229

SE: sensitivity; Sp: specificity; TP: true positive; FP: false positive; FN: false negative; TN: true negative.

### Meta-analysis of diagnostic accuracy estimates

**Fig 2:**
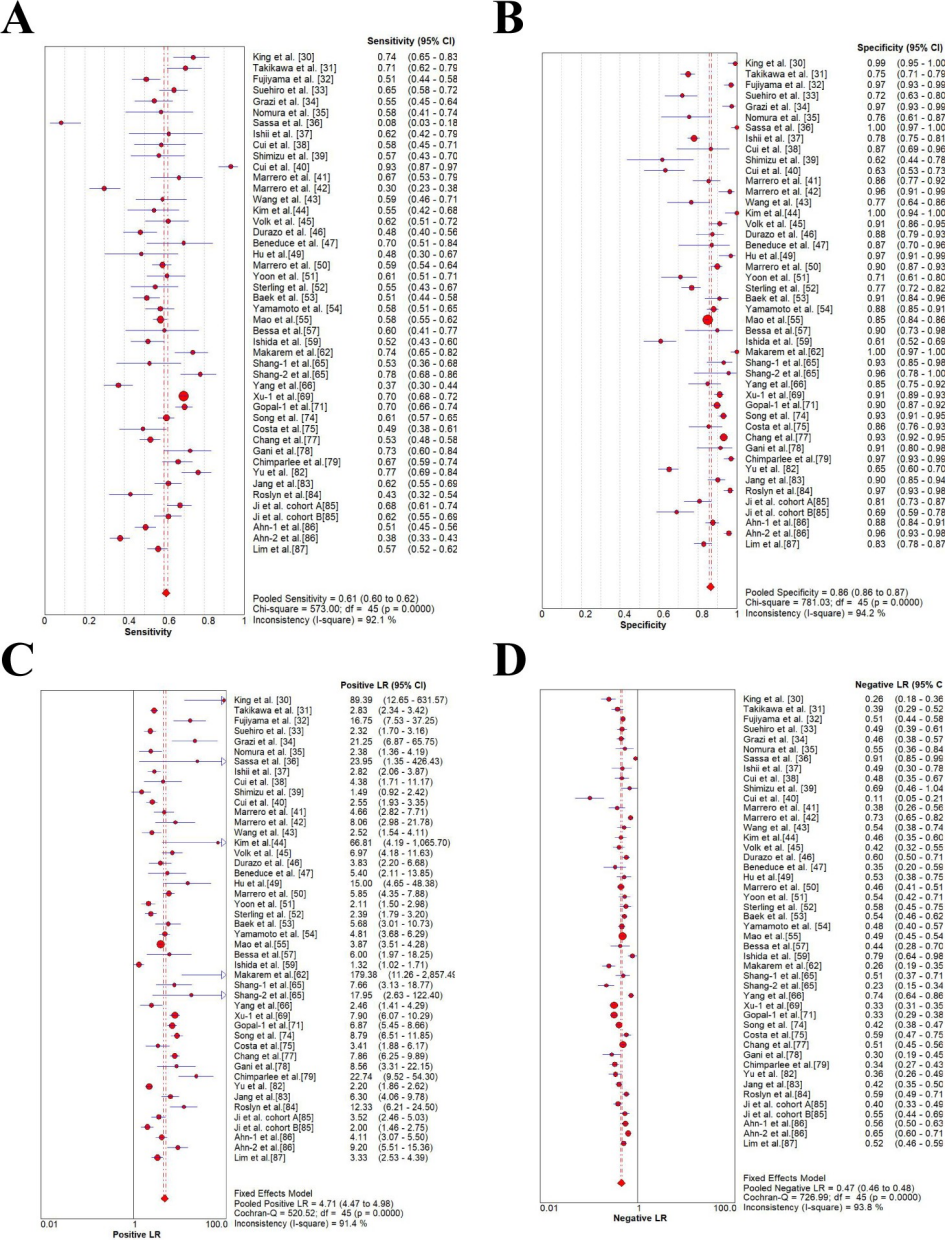
Forest plots of the estimates for AFP in HCC diagnosis (20–100 ng/mL). (A) Pooled sensitivity. (B) Pooled specificity. (C) Pooled positive LR. (D) Pooled negative LR.

**Fig 3:**
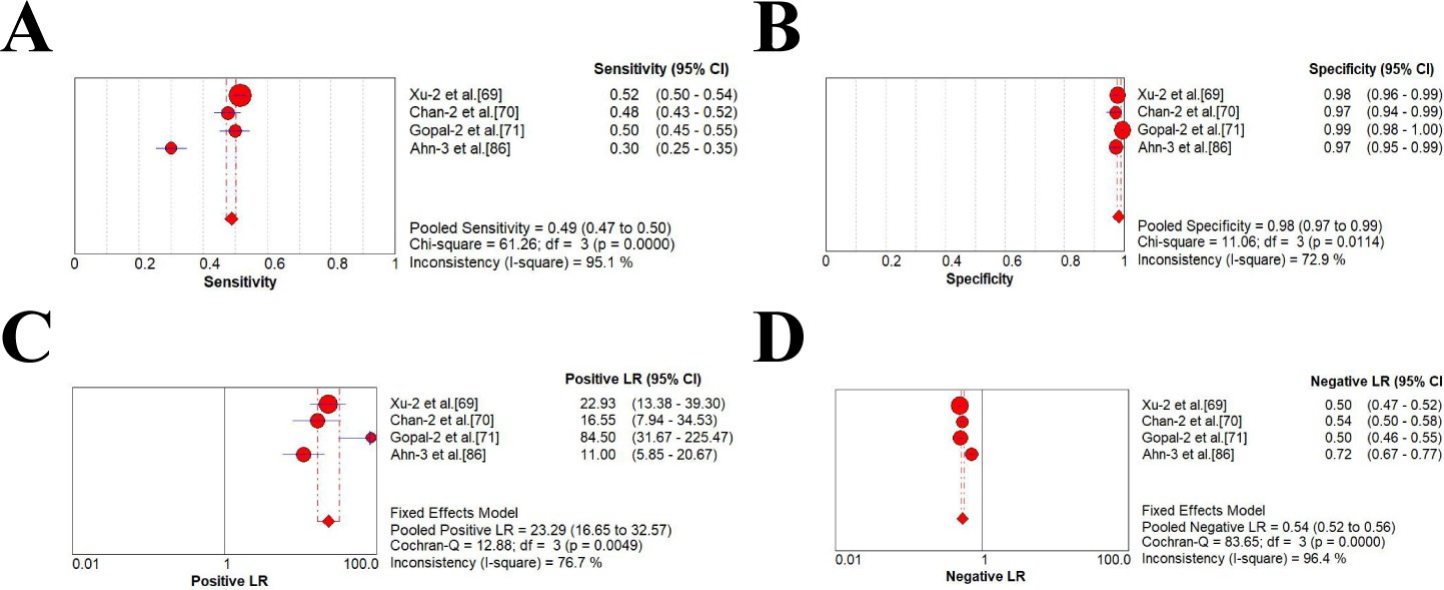
Forest plots of the estimates for AFP in HCC diagnosis (200 ng/mL). (A) Pooled sensitivity. (B) Pooled specificity. (C) Pooled positive LR. (D) Pooled negative LR.

**Fig 4:**
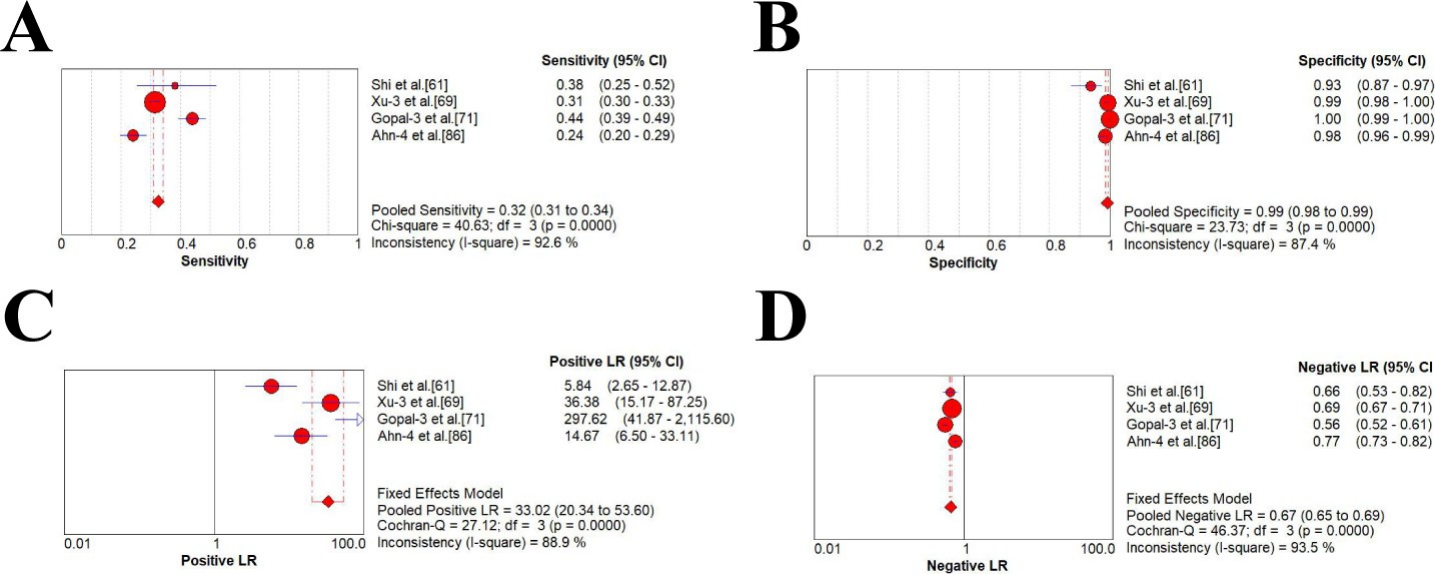
Forest plots of the estimates for AFP in HCC diagnosis (400 ng/mL). (A) Pooled sensitivity. (B) Pooled specificity. (C) Pooled positive LR. (D) Pooled negative LR.

As was shown in **Table 3** and **Figs 2–4**, four studies with AFP threshold of 400 ng/mL showed the summary sensitivity and specificity of 0.32 (95%CI 0.31–0.34) and 0.99 (95%CI 0.98–0.99), respectively, while eighteen studies with 400 ng/mL plus ultrasound showed the pooled sensitivity and specificity of 0.41 (95%CI 0.39–0.43) and 0.94 (95%CI 0.93–0.94), respectively. Four studies with AFP threshold of 200 ng/mL showed the summary sensitivity and specificity of 0.49 (95%CI 0.47–0.50) and 0.98 (95%CI 0.97–0.99), respectively, while eighteen studies with 200 ng/mL plus ultrasound showed the pooled sensitivity and specificity of 0.54 (0.52–0.55) and 0.94 (0.93–0.94), respectively. Forty-six studies with AFP threshold of 20–100 ng/mL showed the summary sensitivity and specificity of 0.61 (95%CI 0.60–0.62) and 0.86 (95%CI 0.86–0.87), respectively, while sixty studies eighteen studies with 20–100 ng/mL plus ultrasound showed the pooled sensitivity and specificity of 0.62 (0.61–0.63) and 0.88 (0.88–0.89), respectively.

**Table 3:** Diagnostic accuracy estimates based on varied thresholds of AFP.

	Cut-off Value (ng/mL)					
	20–100		200		400	
	AFP	AFP+US	AFP	AFP+US	AFP	AFP+US
Sensitivity	0.61 (0.60–0.62)	0.62 (0.61–0.63)	0.49 (0.47–0.50)	0.54 (0.52–0.55)	0.32 (0.31–0.34)	0.41 (0.39–0.43)
Specificity	0.86 (0.86–0.87)	0.88 (0.88–0.89)	0.98 (0.97–0.99)	0.94 (0.93–0.94)	0.99 (0.98–0.99)	0.94 (0.93–0.94)
+LR	4.71 (4.47–4.98)	5.13 (4.89–5.38)	23.29 (16.65–32.57)	13.63 (11.86–15.67)	33.02 (20.34–53.6)	13.28 (11.59–15.23)
-LR	0.47 (0.46–0.48)	0.44 (0.43–0.45)	0.54 (0.52–0.56)	0.43 (0.41–0.45)	0.67 (0.65–0.69)	0.50 (0.48–0.52)
dOR	10.64 (9.91–11.42)	12.25 (11.46–13.10)	42.06 (29.88–59.20)	46.65 (37.62–57.84)	47.63 (29.22–77.64)	50.56 (39.58–64.58)
AUC	0.8330	0.8464	0.9311	0.9359	0.9368	0.9394
SE(AUC)	0.0036	0.0032	0.0084	0.0049	0.0111	0.0054
Q*	0.7654	0.7778	0.8664	0.8723	0.8734	0.8767
SE(Q*)	0.0033	0.0030	0.0101	0.0061	0.0138	0.0068

AFP alpha-fetoprotein, US ultrasound, +LR positive likelihood ratio, -LR negative likelihood ratio, dOR diagnostic odds ratio, AUC area under curve, SE standard error

The result from AFP alone as the marker indicated that the specificity of the threshold 400 ng/mL was the highest (99.0%), but the sensitivity was the lowest (32.0%). The specificity of the 200 ng/mL was 1.0% lower than that of the 400 ng/mL, but the sensitivity could increase to 49.0%, with dOR being the highest (42.06%). The threshold of 20–100 ng/mL owned the greatest sensitivity of 61.0%, but the specificity and dOR were lower than that of 200 ng/mL and 400 ng/mL.

### Threshold identification by SROC analysis

**Fig 5:**
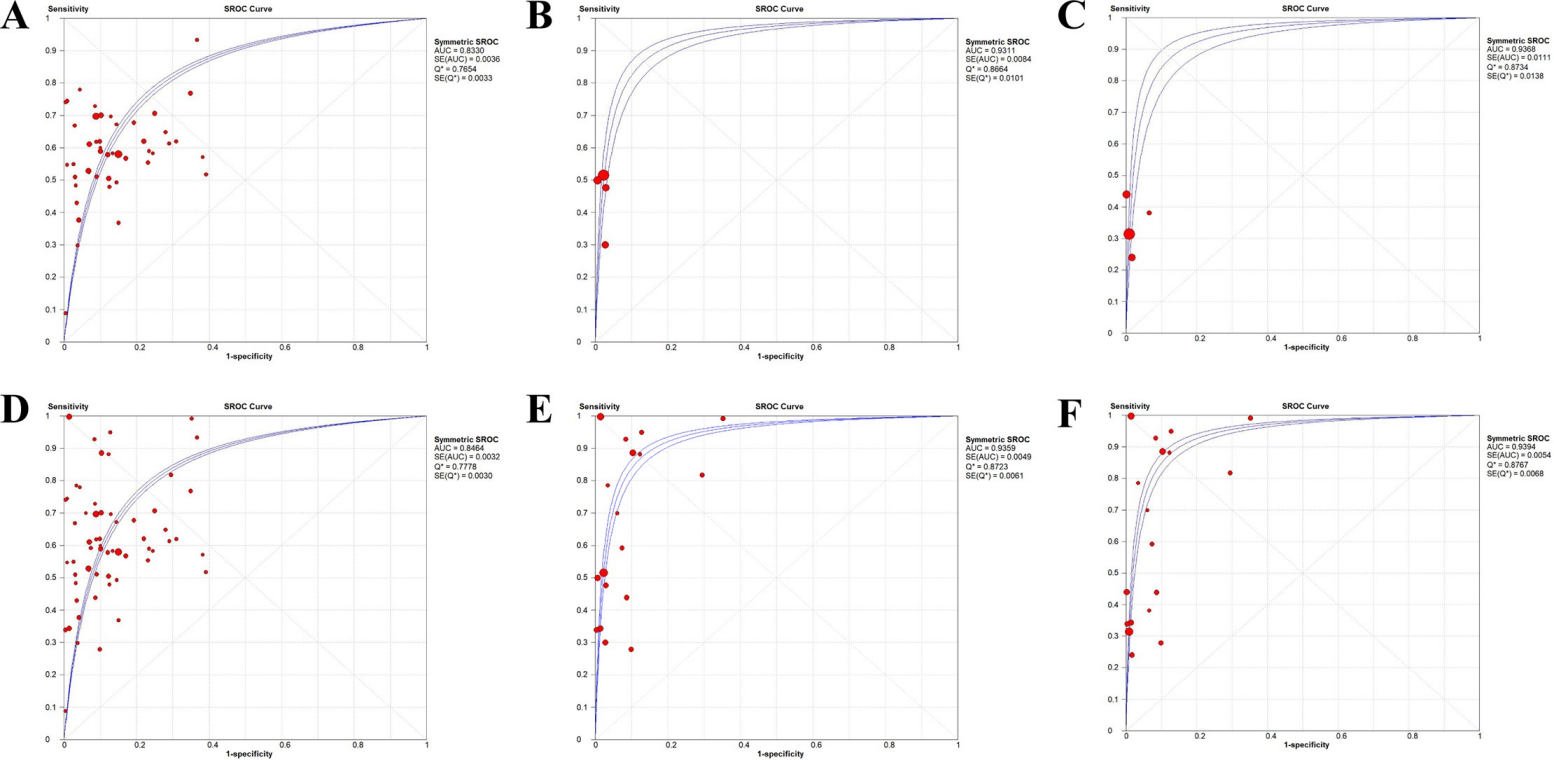
Summary receiver operating characteristic curves (SROC). (A). SROC curve for AFP in 20–100 ng/mL. (B). SROC curve for AFP in 200 ng/mL. (C). SROC curve for AFP in 400 ng/mL. (D) SROC curve for AFP in 20–100 ng/mL combined with ultrasound. (E) SROC curve for AFP in 200 ng/mL combined with ultrasound. (F) SROC curve for AFP in 400 ng/mL combined with ultrasound.

As is shown in **Table 3** and **Fig 5**, The AUC of SROC and Q index of 400 ng/mL threshold were 0.9368 and 0.8734, respectively, which were significantly higher than those in 200 ng/mL threshold (0.9311 and 0.8664, respectively) and higher than those in 20-100ng/mL threshold (0.8330 and 0.7654, respectively). Similarly, when combined with ultrasound, the AUC of SROC and Q index of 400 ng/mL threshold were 0.9394 and 0.8767, respectively, which were significantly higher than those in 200 ng/mL threshold (0.9359 and 0.8723, respectively) and higher than those in 20–100 ng/mL threshold (0.8464 and 0.7778, respectively).

### Heterogeneity test and meta-regression analysis

There was no heterogeneity between groups of different threshold (p > 0.05), as was shown in **Table 4**. However, there existed heterogeneity in sensitivity, specificity, + LR, -LR and dOR within groups with varied threshold, as was shown in **Table 5**. This heterogeneity may be related to the diversity of population selection, including hepatitis B (HBV) and hepatitis C (HCV), as well as some mixed cases, along with diverse detection methods, instruments, reagents, standards. However, only indicators of potential heterogeneity sources such as control, year, country, sample type, assay type and etiology (HBV, HCV or MIX) could be extracted from the included articles. The P-value > 0.10 was realized as homogeneous [88], and no statistically significant effect existed on heterogeneity of three groups (P > 0.10), as shown in **Table 6**.

**Table 4:** Spearman correlation analysis results.

	Cut-off Value (ng/mL)					
	20–100		200		400	
	AFP	AFP+US	AFP	AFP+US	AFP	AFP+US
Rs	0.22	0.235	-0.6	0.482	-0.4	0.515
p value	0.142	0.071	0.4	0.043	0.6	0.029

AFP alpha-fetoprotein, US ultrasound, Rs rank correlation spearman

**Table 5:** Chi-square test and Cochrane-Q test results.

	Cut-off Value (ng/mL)					
	20–100		200		400	
	AFP	AFP+US	AFP	AFP+US	AFP	AFP+US
**Sensitivity**						
X^2^	573	1020.46	61.26	648.00	40.63	937.09
p value	<0.0001	<0.0001	<0.0001	<0.0001	<0.0001	<0.0001
**Specificity**						
X^2^	781.03	1559.36	11.06	738.36	23.73	783.16
p value	<0.0001	<0.0001	0.0114	<0.0001	<0.0001	<0.0001
**+LR**						
Cochrane-Q	520.52	934.42	12.88	737.99	27.12	716.93
p value	<0.0001	<0.0001	0.0049	<0.0001	<0.0001	<0.0001
**-LR**						
Cochrane-Q	726.99	968.77	83.65	431.24	46.37	864.11
p value	<0.0001	<0.0001	<0.0001	<0.0001	<0.0001	<0.0001
**dOR**						
Cochrane-Q	315.91	460.09	16.95	127.94	22.75	138.79
p value	<0.0001	<0.0001	0.0007	<0.0001	<0.0001	<0.0001

+LR positive likelihood ratio, -LR negative likelihood ratio, dOR diagnostic odds ratio

**Table 6:** Meta-regression analyses of potential source of heterogeneity.

Factors	Coeff.	Std. err.	P-value	RDOR
20–100 ng/ml				
Year	0.077	0.1606	0.6339	1.08
Country	0.062	0.0568	0.2819	1.06
Control	0.195	0.1531	0.2097	1.22
Sample type	0.030	0.2874	0.9169	1.03
Etiology	0.120	0.1410	0.3986	1.13
Assay type	0.022	0.2917	0.9409	1.02
200 ng/ml				
Country	-1.153	0.2972	0.1606	0.32
Control	1.195	0.9167	0.4165	3.3
Etiology	-0.403	0.8508	0.7183	0.67
400 ng/ml				
Country	-0.495	0.5226	0.5173	0.61
Control	1.055	1.3062	0.5676	2.87
Etiology	0.104	0.7493	0.9119	1.11

Coeff coefficient, RDOR ratio of the diagnostic odds ratio.

### Publication bias

Deek’s funnel plot showed a slope coefficient of 3.59 (p = 0.534), -42.60 (p = 0.666), -33.98 (p = 0.691) for included studies with 20–100, 200, 400 ng/mL, respectively, which indicated symmetry in data, where publication bias was not suggestive (**S1–S3 Figs**, online supplement).

## Discussion

The disagreement between different international guidelines in terms of the AFP threshold for HCC diagnosis has been continued for several decades, and it has not yet been revolved so far. This article comprehensively reviewed the evidence for the threshold of AFP, and the results showed that AFP threshold of 400 ng/mL reporting the summary sensitivity of 0.32 (95%CI 0.31–0.34) and specificity of 0.99 (95%CI 0.98–0.99), was better than those of the threshold of 200 ng/mL (sensitivity of 0.49 (95%CI 0.47–0.50) and specificity of 0.98 (95%CI 0.97–0.99)), and better than those of the threshold of 20–100 ng/mL (sensitivity of 0.61 (95%CI 0.60–0.62) and specificity of 0.86 (95%CI 0.86–0.87)). The AUC of SROC and Q index of 400 ng/mL threshold were 0.9368 and 0.8734, respectively, which were significantly higher than those in 200 ng/mL threshold (0.9311 and 0.8664, respectively) and higher than those in 20–100 ng/mL threshold (0.8330 and 0.7654, respectively). Besides, similar result that favored 400 ng/mL were shown in the threshold in terms of AFP combined with ultrasound. The overall result indicated that the application of the AFP threshold of 400 ng/mL should be recommended for the diagnosis of HCC no matter it is used alone or combined with ultrasound to monitor the HCC.

It is well established that AFP level has been an optimal diagnostic marker for early diagnosis of HCC because of its well performance of sensitivity and specificity. However, along with HCC, there are other tumor contributors to the rise of AFP levels, such as reproductive system tumors; besides, the process of liver cell regeneration after an acute inflammation could also lead to the occurrence of a sharp increase in AFP levels during the progress of chronic liver diseases like hepatitis and liver cirrhosis[89–91]. Therefore, further laboratory examinations and imaging tests should be provided to combine the result of AFP to make a definite diagnosis [92, 93]. Because of this, the AFP threshold for the diagnosis of HCC is still controversial. AFP ≥ 400 ng/mL is recommended as the diagnostic criteria of HCC in the Chinese guideline for diagnosis and treatment of primary liver cancer (2017 edition) [94]. Nevertheless, Cedrone et al. [95] reported that the level of AFP in patients who had HCC was not affected by HBV or HCV, and a better threshold of serum AFP level should be 50 ng/mL. Another voice from Xu Jianye et al. [96] proposed that the 150 ng/mL diagnostic threshold of AFP for HCC showed better efficacy. Moreover, Zhang Jianhua et al. [20] proved that a low concentration of AFP in the range of 20–200 ng/mL could be used for early screening in the high risk population which could also be combined with ultrasound. However, the 2011 American Society of Hepatology HCC guidelines no longer use AFP as a screening method for HCC [97]. But what should draw our great attentions is the fact that unlike American, the major cause of HCC in other countries such as China is viral hepatitis, so that the dynamic surveillance of AFP level along with ultrasound in the screening among HCC high-risk population [98] still owns its great clinical application [99, 100]. What should actually be addressed in the next version guidelines of America, Europe, Asian-Pacific, and China, is the threshold of AFP in different phase in HCC management.

This meta analysis has its strengths and limitations. This systematic review included 59 articles and a total of 11,731 HCC cases and 21,972 non-HCC cases, which has summarized the evidence from the largest number of researches and participants representative of varied population from all over the world up to now. All the positive and negative cases in this review were confirmed by histomorphology, which ruled out the misclassification bias, and the quality of the included researches showed a low risk of bias. However, there is not without limitations. The articles in this meta analysis was restricted to the publications only in English language, which might missed the studies published in other languages. What is worth mentioning, in this review there are 20,732 cases from Asia, 630 cases from Africa, 5,924 cases from Europe, 8,666 cases from North America, which means that there might be selection bias when giving the conclusion of this article to the whole population; however, the results from meta-regression to detect the heterogeneity sources did not find any significant difference between countries. Furthermore, we have also detected considerable heterogeneity between three groups of varied threshold, and the meta-regression model has not discovered any heterogeneity resource with statistical significance. There also exists potential imbalance between the three groups of different threshold in terms of the number of the studies in each threshold group.

In conclusion, the present meta analysis suggests that AFP levels show good accuracy in HCC diagnosis, and the threshold of AFP with 400 ng/mL is better than that of 200 ng/mL and 20–100 ng/mL in terms of sensitivity and specificity no matter AFP is used alone or combined with ultrasound. Although included studies showed a low risk of bias, and publication bias was not suggestive, yet heterogeneity existed within groups, which might lead to the different threshold across geographic regions. Despite the current conclusion that AFP threshold of 400 ng/mL should be used for the diagnosis of HCC, the threshold of 20 ng/mL should also be suggested to lead to the decision to let a patient go into the surveillance program for HCC due to its high sensitivity. Future studies should pay more attention to the dynamic change of AFP along with the advance of HCC, where artificial intelligence might be applied to construct a model to predict the prognosis of HCC.

## Materials and methods

This systematic review was performed according to the MOOSE and reported in accordance with PRISMA statement [27, 28]. The protocol was registered at PROSPERO (CRD42019133742, http://www.crd.york.ac.uk/PROSPERO).

### Search strategy and article screening

The Medline and EMBASE databases were searched from inception up to November 2019 with the following terms: "alpha-Fetoproteins or AFP" AND "Carcinoma, Hepatocellular or Hepatocellular Carcinomas or Liver Cell Carcinoma" (The detailed search strategy was described in **S1 Table** and **S2 Table**). Besides, we reviewed the references in identified projects for further potential studies. Two reviewers independently screened the titles and abstracts of all retrieved records to find potentially appropriate studies, and then by reading the full text they evaluated the remaining records to identify studies suitable for data synthesis. Any disagreement was resolved by consensus or arbitrator.

### Inclusion criteria

We finally included original articles that met the following criteria:

1. Type of the study was diagnostic accuracy study.
2. Participants in the study included both the patients with HCC diagnosed by pathological diagnosis (gold standard) were taken as the case group and the patients with clinically diagnosed non-liver cancer as the control group.
3. Indicators to be evaluated in the study included AFP.
4. There was a definite AFP measurement value in the article.
5. Complete diagnostic four-grid table data could be obtained from the literature. (the indicators for HCC diagnosis should be directly or indirectly calculated or extracted, including true negative (TN) value, false negative (FN) value, false positive (FP)value, the true positive (TP) value, specificity, and sensitivity)

### Exclusion criteria

1. Non-English published studies.
2. Conference abstracts, reviews, comments, opinions, letters, and editorials.
3. Case reports, biochemical and experimental studies.
4. The sample detected in the study was not plasma or serum.

### Information extraction and quality assessment

Basic information of each included studies was extracted by two reviewers independently. The QUADAS-2 was used to evaluate the quality of diagnostic test literature by two reviewers independently [29]. The evaluation tool includes three aspects—variation, bias, and report quality—and eleven items, where the answer of each item consists of three choices: "Yes," "No," and "unclear." "Yes" means the study meet the criterion, "No" means not satisfied or not mentioned, and "not clear" is partially satisfied or unable to obtain sufficient information from the literature.

### Data extraction and statistical processing

The diagnostic four-grid table data including TN, FN, FP, and TP were extracted from the included literatures, and Meta Disc 1.4 as well as Stata 15.0 software were used for statistical processing. The random effect model was applied to summarize the accuracy estimates if there was heterogeneity, while the fixed-effect model was applied if there was not. We calculated summary estimates of sensitivity, specificity, diagnostic odds ratio (dOR), positive likelihood ratio (+ LR), negative likelihood ratio (- LR). A summary receiver operating characteristic (SROC) curve was also displayed and the area under curve (AUC), and Q * index was used to determine the threshold. Meta-analysis was used to obtain the combined value of the accuracy indicators and their 95%CI, The test level is α = 0.05. The heterogeneity caused by threshold effect was examined by Spearman correlation analysis, and sensitivity and specificity heterogeneity was examined by the chi-square test. The -LR and + LR were examined by Cochrane-Q test. Meta-regression analysis was used to detect the contributors of the heterogeneity. Deek’s funnel plot was used to assess the publication bias, and a slope coefficient with p <0.10 revealed significant bias.

## Supporting information

S1 Prisma PRISMA-P (Preferred reporting items for systematic review and meta-analysis protocols) 2015 checklist: Recommended items to address in a systematic review protocol*. (DOC)

S1 Table Search strategy used in Medline. (DOCX)

S2 Table Search Strategy Used in Embase. (DOCX)

S3 Table Evaluation of quality of included studies using the QUADAS-2 tool. (DOCX)

S1 Fig Deek’s funnel plot (20–100 ng/mL). (TIF)

S2 Fig Deek’s funnel plot (200 ng/mL). (TIF)

S3 Fig Deek’s funnel plot (400 ng/mL). (TIF)
