# Supplementary material for: The threshold of alpha-fetoprotein (AFP) for the diagnosis of hepatocellular carcinoma: A systematic review and meta-analysis
Source: PLoS One. 2020 Feb 13;15(2):e0228857. doi: 10.1371/journal.pone.0228857 (PMC7018038; doi:10.1371/journal.pone.0228857)
Supplement: S1 Table — (DOCX) [file pone.0228857.s002.docx]

**Supplementary Table 1. Search Strategy Used in PubMed, November, 2109**

| Number | Search Items | Items Found |
| --- | --- | --- |
| 1 | (((((((alpha-Fetoproteins[MeSH Terms]) OR alpha-Fetoproteins[Title/Abstract]) OR “alpha Fetoproteins”[Title/Abstract]) OR alpha-Fetoprotein[Title/Abstract]) OR “alpha Fetoprotein”[Title/Abstract]) OR “alpha fetal protein”[Title/Abstract]) OR alpha-fetal-protein[Title/Abstract]) OR AFP[Title/Abstract] | 27,443 |
| 2 | ((((((((((((”Carcinoma, Hepatocellular”[MeSH Terms]) OR ”Carcinoma, Hepatocellular”[Title/Abstract]) OR “Hepatocellular Carcinomas”[Title/Abstract]) OR “Hepatocellular Carcinoma”[Title/Abstract]) OR “Carcinomas, Hepatocellular”[Title/Abstract]) OR “Liver Cell Carcinoma”[Title/Abstract]) OR “Liver Cell Carcinomas”[Title/Abstract]) OR Hepatoma[Title/Abstract]) OR Hepatomas[Title/Abstract]) OR “Carcinoma, Liver Cell”[Title/Abstract]) OR “Carcinomas, Liver Cell”[Title/Abstract]) OR “Cell Carcinoma, Liver”[Title/Abstract]) OR “Cell Carcinomas, Liver”[Title/Abstract] | 126,895 |
| 3 | Numbers 1–2 | 10,269 |
