# Supplementary material for: The threshold of alpha-fetoprotein (AFP) for the diagnosis of hepatocellular carcinoma: A systematic review and meta-analysis
Source: PLoS One. 2020 Feb 13;15(2):e0228857. doi: 10.1371/journal.pone.0228857 (PMC7018038; doi:10.1371/journal.pone.0228857)
Supplement: S2 Table — (DOCX) [file pone.0228857.s003.docx]

**Supplementary Table 2. Search Strategy Used in Embase, November, 2109**

| Number | Search Items | Items Found |
| --- | --- | --- |
| 1 | 'alpha fetoprotein'/exp OR 'alpha fetoprotein':ti,ab,kw OR 'alpha fetoprotein blood level':ti,ab,kw OR 'afp':ti,ab,kw | 45,017 |
| 2 | 'liver cell carcinoma'/exp OR 'liver cell carcinoma':ti,ab,kw OR 'liver cancer':ti,ab,kw OR 'liver tumor':ti,ab,kw OR 'hepatocellular carcinoma':ti,ab,kw | 188,709 |
| 3 | Numbers 1–2 | 19,559 |
