# Supplementary material for: The threshold of alpha-fetoprotein (AFP) for the diagnosis of hepatocellular carcinoma: A systematic review and meta-analysis
Source: PLoS One. 2020 Feb 13;15(2):e0228857. doi: 10.1371/journal.pone.0228857 (PMC7018038; doi:10.1371/journal.pone.0228857)
Supplement: S3 Table — (DOCX) [file pone.0228857.s004.docx]

**Supplementary Table 3. Evaluation of quality of included studies using the QUADAS tool**

|  | 1 | 2 | 3 | 4 | 5 | 6 | 7 | 8 | 9 | 10 | 11 | 12 | 13 | 14 |
| --- | --- | --- | --- | --- | --- | --- | --- | --- | --- | --- | --- | --- | --- | --- |
| King et al. ^[30]^ | Y | Y | Y | Y | Y | Y | U | Y | Y | Y | Y | N | N | N |
| Takikawa et al. ^[31]^ | Y | Y | Y | Y | Y | Y | U | Y | Y | Y | Y | N | N | N |
| Fujiyama et al. ^[32]^ | Y | Y | Y | Y | Y | Y | U | Y | Y | Y | Y | N | N | N |
| Suehiro et al. ^[33]^ | Y | Y | Y | Y | Y | Y | U | Y | Y | Y | Y | N | N | N |
| Grazi et al. ^[34]^ | Y | Y | Y | Y | Y | Y | U | Y | Y | Y | Y | N | N | N |
| Nomura et al. ^[35]^ | Y | Y | Y | Y | Y | Y | U | Y | Y | Y | Y | N | N | N |
| Sassa et al. ^[36]^ | Y | Y | Y | Y | Y | Y | U | Y | Y | Y | Y | N | N | N |
| Ishii et al. ^[37]^ | Y | Y | Y | Y | Y | Y | U | Y | Y | Y | Y | N | N | N |
| Cui et al. ^[38]^ | Y | Y | Y | Y | Y | Y | U | Y | Y | Y | Y | N | N | N |
| Shimizu et al. ^[39]^ | Y | Y | Y | Y | Y | Y | U | Y | Y | Y | Y | N | N | N |
| Cui et al. ^[40]^ | Y | Y | Y | Y | Y | Y | U | Y | Y | Y | Y | N | N | N |
| Marrero et al. ^[41]^ | Y | Y | Y | Y | Y | Y | U | Y | Y | Y | Y | N | N | N |
| Marrero et al. ^[42]^ | Y | Y | Y | Y | Y | Y | U | Y | Y | Y | Y | N | N | N |
| Wang et al. ^[43]^ | Y | Y | Y | Y | Y | Y | U | Y | Y | Y | Y | N | N | N |
| Kim et al.^[44]^ | Y | Y | Y | Y | Y | U | U | N | Y | Y | Y | N | Y | N |
| Volk et al. ^[45]^ | Y | Y | Y | Y | Y | Y | U | Y | Y | Y | Y | N | N | N |
| Durazo et al. ^[46]^ | Y | Y | Y | Y | Y | Y | U | Y | Y | Y | Y | N | N | N |
| Beneduce et al. ^[47]^ | Y | Y | Y | Y | Y | Y | U | Y | Y | Y | Y | N | N | N |
| Wang et al.^[48]^ | Y | Y | Y | Y | Y | Y | U | Y | Y | Y | Y | N | N | N |
| Hu et al.^[49]^ | Y | Y | Y | Y | Y | Y | U | Y | Y | Y | Y | N | N | N |
| Marrero et al. ^[50]^ | Y | Y | Y | Y | Y | Y | U | Y | Y | Y | Y | N | N | N |
| Yoon et al. ^[51]^ | Y | Y | Y | Y | Y | Y | U | Y | Y | Y | Y | N | N | N |
| Sterling et al. ^[52]^ | Y | Y | Y | Y | Y | Y | U | Y | Y | Y | Y | N | N | N |
| Baek et al. ^[53]^ | Y | Y | Y | Y | Y | Y | U | Y | Y | Y | Y | N | N | N |
| Yamamoto et al. ^[54]^ | Y | Y | Y | Y | Y | Y | U | Y | Y | Y | Y | N | N | N |
| Mao et al.^[55]^ | Y | Y | Y | Y | Y | Y | U | Y | Y | Y | Y | N | N | N |
| Ozkan et al.^[56]^ | Y | Y | Y | Y | Y | Y | U | Y | Y | Y | Y | N | N | N |
| Bessa et al.^[57]^ | Y | Y | Y | Y | Y | Y | U | Y | Y | Y | Y | N | N | N |
| Sharma et al. ^[58]^ | Y | Y | Y | Y | Y | Y | U | Y | Y | Y | Y | N | N | N |
| Ishida et al. ^[59]^ | Y | Y | Y | Y | Y | Y | U | Y | Y | Y | Y | N | N | N |
| Tian et al.^[60]^ | Y | Y | Y | Y | Y | Y | U | Y | Y | Y | Y | N | N | N |
| Shi et al.^[61]^ | Y | Y | Y | Y | Y | Y | U | Y | Y | Y | Y | N | N | N |
| Makarem et al.^[62]^ | Y | Y | Y | Y | Y | Y | U | N | Y | Y | Y | N | N | N |
| Morota et al. ^[63]^ | Y | Y | Y | Y | Y | Y | U | Y | Y | Y | Y | N | N | N |
| Salem et al.^[64]^ | Y | Y | Y | Y | Y | Y | U | N | Y | Y | Y | N | N | N |
| Shang et al.^[65]^ | Y | Y | Y | Y | Y | U | U | Y | Y | N | Y | N | N | N |
| Yang et al.^[66]^ | Y | Y | Y | Y | Y | Y | U | N | Y | Y | Y | N | Y | N |
| Choi et al. ^[67]^ | Y | Y | Y | Y | Y | Y | U | Y | Y | Y | Y | N | N | N |
| Ertle et al. ^[68]^ | Y | Y | Y | Y | Y | Y | U | Y | Y | Y | Y | N | N | N |
| Xu et al. ^[69]^ | Y | Y | Y | Y | Y | Y | U | Y | Y | Y | Y | N | N | N |
| Chan et al. ^[70]^ | Y | Y | Y | Y | Y | Y | U | Y | Y | Y | Y | N | N | N |
| Gopal et al. ^[71]^ | Y | Y | Y | Y | Y | Y | U | Y | Y | Y | Y | N | N | N |
| Lee et al.^[72]^ | Y | Y | Y | Y | U | Y | U | Y | Y | N | Y | N | U | N |
| Nabih et al.^[73]^ | Y | Y | Y | Y | Y | Y | U | Y | Y | Y | Y | N | Y | N |
| Song et al. ^[74]^ | Y | Y | Y | Y | Y | Y | U | Y | Y | Y | Y | N | N | N |
| Costa et al.^[75]^ | Y | Y | Y | U | Y | Y | U | Y | Y | Y | Y | N | U | N |
| Poté et al. ^[76]^ | Y | Y | Y | Y | Y | Y | U | Y | Y | Y | Y | N | N | N |
| Chang et al.^[77]^ | Y | Y | Y | Y | Y | Y | U | Y | Y | Y | Y | N | N | N |
| Gani et al.^[78]^ | Y | Y | Y | Y | Y | Y | U | Y | Y | Y | Y | N | N | N |
| Chimparlee et al.^[79]^ | Y | Y | Y | U | Y | Y | U | Y | Y | N | Y | N | U | N |
| Fouad et al.^[80]^ | Y | U | Y | Y | Y | Y | U | Y | Y | Y | Y | U | N | N |
| Ge et al.^[81]^ | Y | Y | Y | Y | U | Y | U | Y | Y | N | Y | N | U | N |
| Yu et al. ^[82]^ | Y | Y | Y | Y | Y | Y | U | Y | Y | Y | Y | N | N | N |
| Jang et al.^[83]^ | Y | Y | Y | Y | Y | Y | U | Y | Y | Y | Y | N | N | N |
| Roslyn et al.^[84]^ | Y | Y | Y | Y | Y | Y | U | Y | Y | N | N | N | N | N |
| Ji et al. ^[85]^ | Y | Y | Y | Y | Y | Y | U | Y | Y | Y | Y | N | N | N |
| Ahn et al. ^[86]^ | Y | Y | Y | Y | Y | Y | U | Y | Y | Y | Y | N | N | N |
| Lim et al. ^[87]^ | Y | Y | Y | Y | Y | Y | U | Y | Y | Y | Y | N | N | N |

QUADAS: quality assessment of diagnostic studies; Y: yes; N: no; U: unclear;

1. Was a consecutive or random sample of patients enrolled?

2. Was a case–control design avoided?

3. Did the study avoid inappropriate exclusions?

4. Were the index test results interpreted without knowledge of the results of the reference standard?

5. If a threshold was used, was it prespecified?

6. Is the reference standard likely to correctly classify the target condition?

7. Were the reference standard results interpreted without knowledge of the results of the index test?

8. Was there an appropriate interval between index tests and reference standard?

9. Did all patients receive a reference standard?

10. Did all patients receive the same reference standard?

11. Were all patients included in the analysis?

12. Are there concerns that the included patients do not match the review question?

13. Are there concerns that the index test, its conduct, or its interpretation differ from the review question?

14. Are there concerns that the target condition as defined by the reference standard does not match the review question?
