# Supplementary figures and images for: The threshold of alpha-fetoprotein (AFP) for the diagnosis of hepatocellular carcinoma: A systematic review and meta-analysis
Source: PLoS One. 2020 Feb 13;15(2):e0228857. doi: 10.1371/journal.pone.0228857 (PMC7018038; doi:10.1371/journal.pone.0228857)

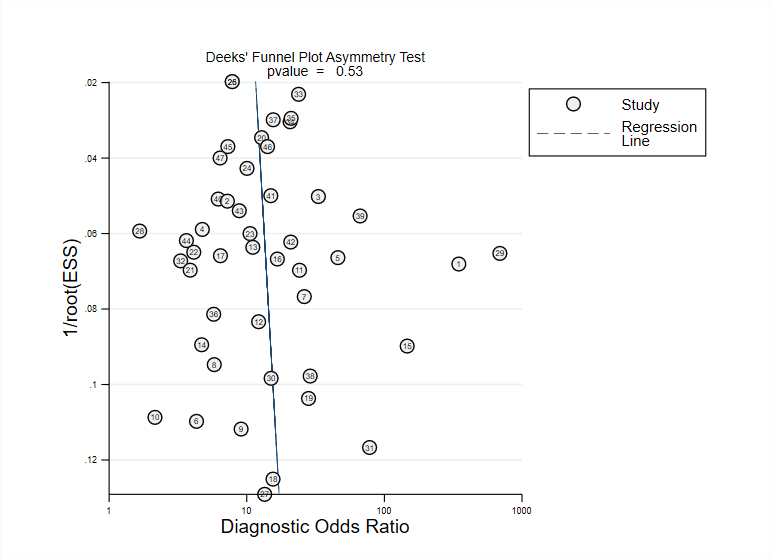

Supplement: S1 Fig — (TIF) [file pone.0228857.s005.tif]

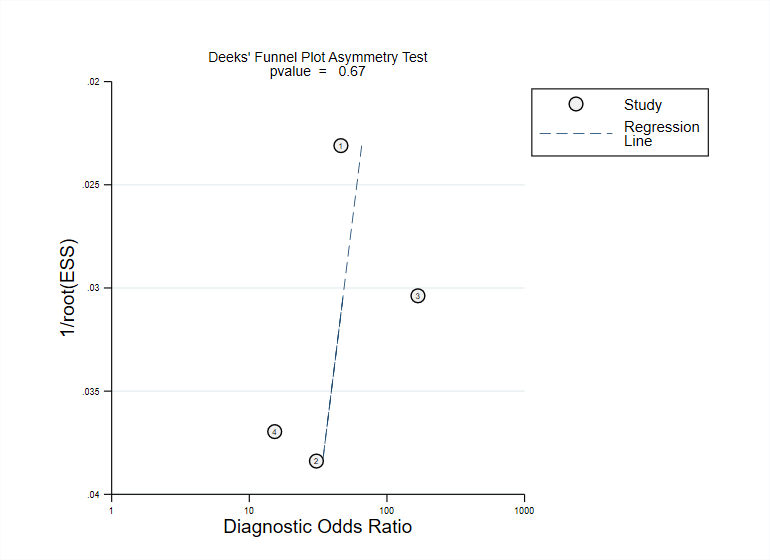

Supplement: S2 Fig — (TIF) [file pone.0228857.s006.tif]

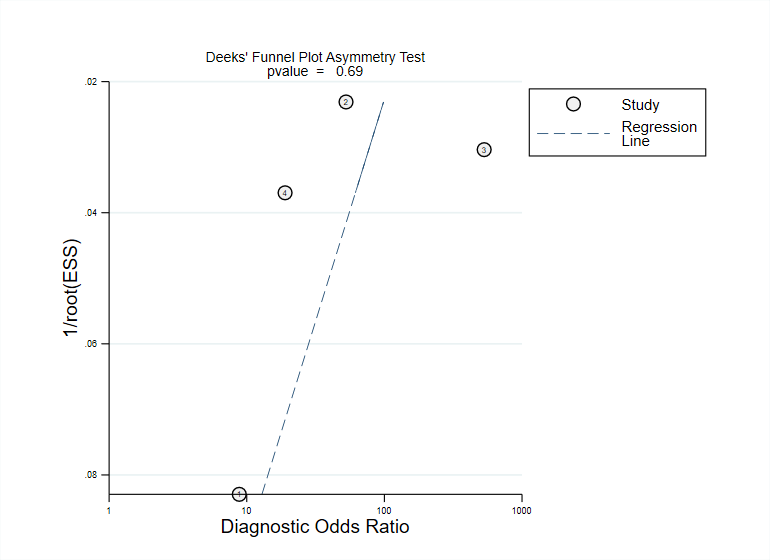

Supplement: S3 Fig — (TIF) [file pone.0228857.s007.tif]
